# Effect of nanoencapsulation on volatile constituents, and antioxidant and anticancer activities of Algerian *Origanum glandulosum* Desf. essential oil

**DOI:** 10.1038/s41598-020-59686-w

**Published:** 2020-02-18

**Authors:** Hatem Ali, Abdel Rahman Al-Khalifa, Abdelhakim Aouf, Habiba Boukhebti, Amr Farouk

**Affiliations:** 1 Food Science and Nutrition Department College of Food Science and Agriculture, King Saud University, Riyadh, Saudi Arabia; 2 Food Technology Department, National Research Center, Cairo, Egypt; 3 Department of Microbiology, Faculty of Sciences of Nature and Life, Ferhat Abbas University, Setif-1, Setif, Algeria; 4 Department of Biology and Plant Ecology, Faculty of Sciences of Nature and Life, Ferhat Abbas University, Setif-1, Setif, Algeria; 5 Flavour and Aroma Chemistry Department, National Research Center, Cairo, Egypt

**Keywords:** Biological techniques, Cancer, Cell biology, Chemical biology, Chemistry, Nanoscience and technology

## Abstract

Nanoencapsulation is an attractive novel technique used for incorporating essential oils in food preparations and pharmaceutical formulae. This study investigated the effect of nanoencapsulation on the composition of volatile compounds, as well as the antioxidant and anticancer activities of hydrodistilled (HD) *Origanum glandulosum* Desf. Oil, which was encapsulated into nanocapsules via High Speed Homogenization (HSH) and into nanoemulsions through High Pressure Homogenization (HPH). Thirty-two volatile components were identified using Gas Chromatography-Mass Spectrometry analysis (GC-MS) in HD essential oil representing 99.04% of the total oil content. GC-MS analysis showed that the use of HPH to prepare nanoemulsions negatively affected the active compounds present in HD oil, particularly carvacrol and thymol, whereas the use of HSH led to significant quantitative differences in the composition of volatiles between HD oil and nanocapsules but generated the same profile. Consistent with the differences in total phenolics, total flavonoids, and volatiles identified in HD and nanoparticles, HD essential oil exhibited a higher antioxidant activity (IC_50_ 4.22 mg/mL) than nanocapsules (IC_50_ 57.51 mg/mL) and nanoemulsion (IC_50_ 78.50 mg/mL), while nanocapsules showed the strongest cytotoxic effect on liver cancer cell line Hep-G2 (54.93 μg/mL) in comparison to HD oil (73.13 μg/mL) and nanoemulsions (131.6 μg/mL).

## Introduction

The *Origanum* genus (Lamiaceae family) includes approximately 38 species that have been studied extensively for potential importance, and uses in flavoring foods and traditional medicine due to their pharmacological characteristics^1^. *Origanum* species are widely distributed in North Africa, eastern Mediterranean, and Siberian regions. According to Kokkini^2^, *Origanum* taxa are rich in essential oils that exhibit well-known antioxidant and antimicrobial properties. Based on the chemical composition of essential oils, *Origanum* species have been classified into three main chemotypes: thymol/carvacrol, linalool/terpinen-4-ol, and sesquiterpenes.

*Origanum glandulosum* Desf., which belong to thymol and /or carvacrol chemotype, is an endemic herb of Algeria, Morocco and Tunisia used in traditional medicine to treat cough, rheumatism, diabetes, and fever^3^. Previous studies have reported antioxidant, antimicrobial, antifungal, and insecticidal activities of the essential oil extracted from *O. glandulosum* Desf^4–7^. However, to the best of our knowledge, the anticancer properties of this oil have not yet been investigated, despite the focus of several recent studies on the use of natural products with potent antioxidant activity in cancer treatment^8^.

Like most essential oils, the use of *O. glandulosum* Desf. oil in food or pharmaceutical industries may have some limitations owing to its aroma, flavor, volatility, poor dispersibility in hydrophilic media, and sensitivity towards oxidation, heat, and light^9^. Nanoencapsulation seems to be an attractive novel approach to avoid these limitations^10^.

Nanoemulsion is one of the nanostructures which can be formulated within the droplet size range of 20–200 nm and has with a higher kinetic stability suggesting that it will exhibit stability during storage. Nanoemulsion, a step towards nanoencapsulation, is formulated through emulsification of oily and aqueous phases using an emulsifier coating molecule^11^. Nanocapsules have been formulated using biodegradable polymers as wall materials, including whey protein^12^, sodium caseinate^13^ and modified starch or maltodextrin^14^.

The above information provides the rationale behind this study that aimed to formulate nanocapsules and nanoemulsion containing *O. glandulosum* Desf. oil. The effects of different nanoencapsulation forms of the essential oil on the volatile constituents, antioxidant activity as well as the cytotoxic effect on liver cancer cell line Hep-G2 were evaluated. Healthy human hepatic cells THLE2 were used as a control to test the putative selectivity of the oil and its nanoparticles. The present study therefore opens perspectives for safer solutions employing compounds of botanical origin associated with nanotechnology for application in both food industry and cancer treatment.

## Methods

### Plant material and chemicals

The aerial parts of *O. glandulosum* Desf. were collected from north Setif (Bougaa), Algeria in July 2018 during the flowering stage. Identification was performed a taxonomist at the laboratory of biology, Faculty of Sciences, Ferhat Abbas University, Setif-1, and the samples were stocked until use. Diethyl ether and methanol were purchased from Fisher Chemicals (Pittsburgh, USA). The mixture of n-alkanes C6–C26, authentic compounds, sodium bicarbonates, linoleic acid (≥99%), Tween 20, Folin-Ciocalteu reagent, 2,2′-diphenyl-1-picrylhydrazyl (DPPH), rutin, aluminium chloride, (+)-catechin, 5-fluorouracil (5-FU), Tetrazolium bromide solution (MTT) and tert-butylhydroquinone were obtained from Sigma Aldrich Chemical Co. (St. Louis, MO, USA). Human hepatocellular carcinoma (HepG2) and normal liver (THLE2) cells were purchased from the VACSERA (Cairo, Egypt). DMSO was provided by Merck, Darmstadt, Germany. Fetal calf serum (FCS) and penicillin/streptomycin were obtained from Hyclone, Logan, UT, USA. Dulbecco’s Modified Eagle Medium (DMEM) was purchased from Gibco; Thermo Fisher Scientific, Inc., Waltham, MA, USA.

### Essential oil extraction by hydrodistillation

The experiment was performed in triplicate. Air dried aerial parts of *O. glandulosum* Desf. (100 g) were cut into small pieces and then subjected to hydrodistillation for 3 h using a Clevenger-type apparatus, following the protocol established by Farouk *et al*.^15^. The extracted essential oils were dried using anhydrous sodium sulfate and stored in airtight glass vials covered with aluminum foil at −20 °C until analysis.

### Preparation of the nanocapsule suspensions and nanoemulsion

Nanoencapsulation of *O. glandulosum* Desf. essential oil was performed using the homogenization technique (Homogenizer PRO 400 PC, SN: 04-01198, U.S.A.) as previously described by Hussein *et al*.^16^ with some modifications. Briefly, sodium alginate was dissolved in distilled water at a concentration of 3 g/100 mL. The solution was left standing for 24 h to disengage bubble before use. One gram of oil and 1% of Tween 20 was added to 100 mL of the polymer solution. The emulsion was created by mixing the solution in a high-speed homogenizer at 18.000 rpm for 20 min. The temperature was maintained at 25 °C. After preparation, samples were stored at 4 °C until use.

The *O. glandulosum* Desf. essential oil-in-water nanoemulsions were prepared using a High-Pressure Homogenization (HPH) technique. Primary emulsions were obtained by mixing a solution consisting of 1 g oil and 1% of Tween 20 in 100 mL of deionized water using an Ultra Turrax T25 (IKA Labortechnik, Germany) at 24000 rpm for 10 min. The primary emulsions were then subjected to HPH in a Nano DeBEE Electric Bench-top Laboratory homogenizer (BEE International, USA) at 350 MPa; the procedure was repeated ten times. Crystallization of the lipid droplets was attained by rapid cooling of the hot nanoemulsions in an ice bath at the end of the HPH processing^17^.

### Gas chromatography–mass spectrometry (GC–MS)

The components of the essential oil obtained using HD and the volatile compounds extracted from nanocapsules and nanoemulsion were analyzed using a GC–MS apparatus. Separation was performed on a Trace GC Ultra Chromatography system (Thermo Scientific, USA) equipped with an ISQ-mass spectrometer (Thermo Scientific, USA) with a 60 m × 0.25 mm × 0.25 μm-thick TG-5MS capillary column (Thermo Scientific, USA). The column separation was programmed at 50 °C with a holding time of 3 min, and then the temperature was increased at a rate of 4 °C per min to 140 °C with a holding time of 5 min. Thereafter, the temperature was increased at a rate of 6 °C per minute to 260 °C for a 5-minisothermal holding time. The injector temperature was 180 °C, the ion source temperature was 200 °C, and the transition line temperature was 250 °C. The carrier gas was helium with a constant flow rate of 1.0 mL/min. The mass spectrometer had a scan range from m/z 40–450, and the ionization energy was set at 70 Ev. The identification of compounds was based on matching profiles with the MS computer library (NIST library, 2005 version) and comparison with authentic compounds and published data^28^. The relative percentage of the identified constituents was calculated from the GC peak areas. Kovat’s index was calculated for each compound, using the retention times of a homologous series of C_6_–C_26_ n-alkanes and by matching with the values reported in literature^5,18,28^.

### Transmission Electron Microscopy (TEM)

The morphology of nanocapsules and naoemulsions was obtained using transmission electron microscopy (JED 1230, JEOL Ltd., and Tokyo, Japan). Twenty microliters of the diluted sample was placed on a film-coated with 200-mesh copper specimen grid for 10 min and the excess fluid was removed by blotting with filter paper. The grids were then stained with one drop of 3% phosphotungstic acid and allowed to dry for 3 min. The coated grids were dried and examined under the TEM microscope. The samples were observed by operating at 160 kV. The average particle size was calculated from TEM images using the ImageJ software^19^.

### Antioxidant activity measurements

#### DPPH radical scavenging assay

Potential antioxidant activity of *O. glandulosum* Desf. oil, nanocapsules and nanoemulsion was assessed according to the methods reported by Hatano *et al*.^20^ in comparison to a synthetic antioxidant used in food industry, tert - butylhydroquinone (TBHQ). The absorbance was measured at 517 nm using a spectrophotometer (Cecil 2010, Cecil Instr. Ltd., Cambridge, UK); All tests were run in triplicate. The arithmetic mean of the results was calculated.

#### Total phenolic content

Total phenolic content of the essential oil, nanocapsules and nanoemulsion was determined using the Folin-Ciocalteu reagent according to a method modified from that of Singleton *et al*.^21^ using gallic acid as the standard. The reaction mixtures were incubated in a thermostat at 45 °C for 45 min before measuring the absorbance at 765 nm.

#### Estimation of flavonoids content

The flavonoid content in samples was determined spectrophotometrically using a method based on the formation of a complex flavonoid–aluminium, having maximum absorptivity at 430 nm. Rutin was used to make the calibration curve. Thereafter, 1 mL of diluted samples were separately mixed with 1 mL of 2% aluminum chloride methanolic solution. After incubation at room temperature for 15 min, the absorbance of the reaction mixture was measured at 430 nm and the flavonoids content was expressed in mg per g of rutin equivalent (RE)^22^.

### Cell culture and evaluation of the cytotoxicity of *O. glandulosum* Desf. oil and its nanoparticles against HepG2 and THLE2 cells by MTT assay

Both HepG2 and THLE2 cells were seeded at a density of 1 × 10^4^ cells/well (100 μl/well) in DMEM medium supplemented with 10% (v/v) FCS and 1% (v/v) penicillin/streptomycin solution and incubated at 37 °C and 5% CO_2_ for 24 hr to obtain 70–80% confluent cultures. The oil and its nanoparticles were applied separately at different concentrations to the wells to achieve final concentrations ranging from 200 to 6.25 μg/ml for HepG2 cells and from 600 to 6.25 μg/ml for THLE2. The positive control wells were supplemented with different concentrations (5–400 µg/mL) of 5-FU. At the end of incubation, 10 μl of 12 mM MTT stock solution (5 mg/ml MTT in sterile PBS) was added to each well. The plate was then incubated for 4 hr at 37 °C. MTT solution was removed and the purple formazan crystal formed at the bottom of the wells was dissolved with 100 μl DMSO for 20 min. A negative control of 10 μl of MTT stock solution was added to 100 μl of medium alone. The absorbance at λ max 570 nm was read on an ELISA reader. The proportion of surviving cells was calculated as (OD of sample − OD of blank)/(OD of control − OD of blank) × 100%. Sigmoidal and dose dependent curves were constructed to plot the results of the experiment. Assays were performed in triplicate. The concentration of the compounds inhibiting 50% of cells (IC_50_) was calculated using the sigmoidal curve^23^.

### Statistical analysis

Statistical analyses were performed using SPSS software version 16. The data were expressed as mean ± SD and analyzed using Student’s t–test and analysis of variance.

## Results and Discussion

### Effect of nanoencapsulation on the volatile oil of *O. glandulosum* Desf

**Figure 1:**
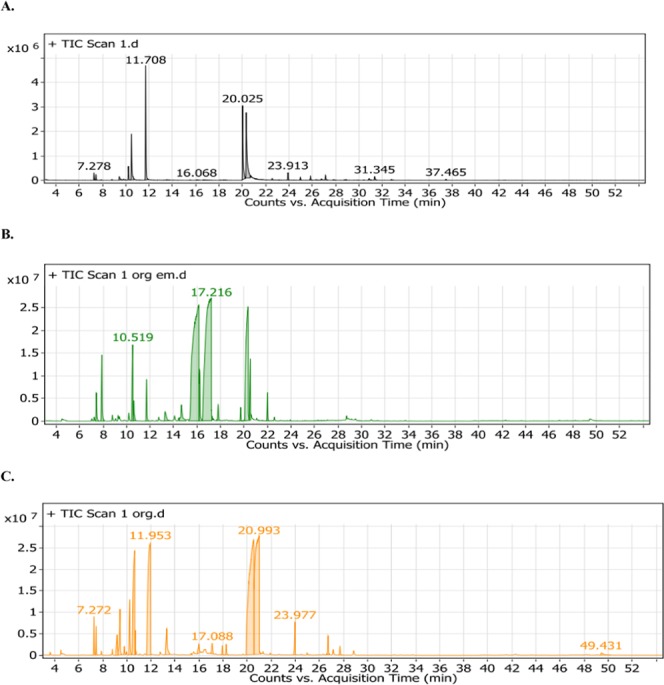
Volatile chromatograms for (**A**) *O. glandulosum* Desf. HD oil, (**B**) nanoemulsion of *O. glandulosum* Desf. oil, and (**C)** nanocapsules of *O. glandulosum* Desf. oil.

The dried aerial parts of *O. glandulosum* Desf. when subjected to hydrodistillation, produced yellowish oil with a yield 1.73 ± 0.06%. These numbers were in agreement with the findings of Belhattab *et al*.^5^. Using GC-MS, a total of 32 compounds, representing 99.04% of the total oil content were identified (Table 1, Fig. 1A). The predominant compounds in the hydrodistilled oil were carvacrol (26.29%), γ-Terpinene (23.43%), Thymol (19.52%), p-cymene (11.67%) and α-Terpinene (3.02%). These findings suggest that the oil belongs to the thymol and /or carvacrol chemotype which is consistent with the findings from previous studies by Sari *et al*.^1^, Mechergui *et al*.^3^ and Belhattab *et al*.^5^.

**Table 1:** Volatile constituents identified from the hydrodistilled (HD), nanocapsules and nanoemulsion of *O. glandulosum* essential oil using GC-MS.

S/N	Compound	KI^a^	% Area^b^ *Origanum glandulosum* Desf. oil			Identification method^c,d^
			HD	Nanoemulsion	Nanocapsules	
1	α-Thujene	928	**1.43 ± 0.12**	n.d.	**1.08 ± 0.07**	MS, KI, & ST
2	α-Pinene	932	**1.13 ± 0.05**	**1.02 ± 0.03**	0.79 ± 0.06	MS, KI, & ST
3	Camphene	971	0.12 ± 0.03	**2.36 ± 0.06**	**1.13 ± 0.04**	MS & KI
4	β–Pinene	978	0.20 ± 0.12	n.d.	n.d.	MS & KI
5	β–Myrcene	991	**1.45 ± 0.13**	n.d.	**2.22 ± 0.06**	MS, KI, & ST
6	α-Phellandrene	998	0.21 ± 0.04	n.d.	0.33 ± 0.08	MS & KI
7	Δ–3-Carene	1001	0.10 ± 0.06	n.d.	**2.63 ± 0.08**	MS & KI
8	α-Terpinene	1004	**3.02 ± 0.08**	n.d.	n.d.	MS, KI, & ST
9	p-Cymene	1008	**11.67 ± 0.33**	**2.86 ± 0.06**	**9.09 ± 0.20**	MS, KI, & ST
10	Limonene	1029	n.d.	0.72 ± 0.07	0.56 ± 0.04	MS & KI
11	γ-Terpinene	1088	**23.43 ± 0.07**	**1.35 ± 0.08**	**15.26 ± 0.14**	MS, KI,& ST
12	Linalool	1089	0.37 ± 0.05	0.73 ± 0.07	**1.8 ± 0.30**	MS & KI
13	Camphor	1141	n.d.	**1.31 ± 0.13**	n.d.	MS & KI
14	Borneol	1148	0.10 ± 0.07	**34.29 ± 0.41**	n.d.	MS & KI
15	Terpinen-4-ol	1155	0.30 ± 0.06	**2.42 ± 0.17**	0.65 ± 0.08	MS & KI
16	α-Terpineol	1165	0.49 ± 0.10	**35.68 ± 0.42**	0.62 ± 0.22	MS & KI
17	Thymol methyl ether	1231	0.19 ± 0.07	n.d.	n.d.	MS & KI
18	Carvacrol methyl ether	1240	0.24 ± 0.08	n.d.	n.d.	MS & KI
19	Thymol	1267	**19.52 ± 0.27**	**13.47 ± 0.26**	**31.81 ± 0.35**	MS, KI, & ST
20	Carvacrol	1276	**26.29 ± 0.15**	**1.93 ± 0.16**	**28.45 ± 0.18**	MS, KI, & ST
21	β–Cubebene	1385	0.33 ± 0.05	n.d.	n.d.	MS & KI
22	β–Caryophyllene	1414	**1.65 ± 0.32**	0.82 ± 0.10	**1.33 ± 0.11**	MS, KI,& ST
23	α-Humulene	1451	0.72 ± 0.17	n.d.	n.d.	MS & KI
24	Germacrene D	1472	**1.12 ± 0.08**	n.d.	n.d.	MS, KI, & ST
25	Bicyclogermacrene	1489	0.14 ± 0.03	n.d.	n.d.	MS & KI
26	α-Muurolene	1491	0.06 ± 0.04	n.d.	n.d.	MS & KI
27	β–Bisabolene	1502	0.52 ± 0.07	n.d.	0.72 ± 0.07	MS & KI
28	γ-Cadinene	1509	**1.27 ± 0.09**	n.d.	n.d.	MS, KI, & ST
29	γ-Bisabolene	1511	0.29 ± 0.07	n.d.	0.36 ± 0.04	MS & KI
30	Spathulenol	1561	0.16 ± 0.08	n.d.	n.d.	MS & KI
31	Caryophyllene oxide	1576	0.23 ± 0.08	n.d.	n.d.	MS & KI
32	α–epi- Muurolol	1638	0.71 ± 0.08	n.d.	n.d.	MS & KI
33	Torreyol	1641	0.14 ± 0.03	n.d.	n.d.	MS & KI
34	α-Cadinol	1649	**1.44 ± 0.10**	n.d.	n.d.	MS, KI, & ST
	Total	—	**99.04**	**98.96**	**98.50**	—

^a^Confirmed by comparison with Kovat’s index on a DB5 column (Adams 2007).

^b^Values represent averages ± standard deviations for triplicate experiments.

^c^Confirmed by comparison with the mass spectrum of the authentic compound.

^d^Identification by comparison with data obtained from the NIST mass spectra library.

n.d: not detected.

The volatiles composition of the oil nanoemulsion was found to be dramatically different from that of the hydrodistilled oil. In the oil nanoemulsion, α-terpineol (35.68%), borneol (34.29%) and Thymol (13.47%) were identified as the major constituents (Table 1, Fig. 1B). Only thirteen compounds were detected in oil nanoemulsion which accounts for 98.69% of the volatile material. Carvacrol, γ-terpinene, p-cymene and many sesquiterpenes were found in very low amounts or not detected at all. Several recent studies have investigated the formulation of nanoemulsion of essential oils and their biological activities. However, very few studies have evaluated how the encapsulation changes the essential oil constituents and consequently the biological activities of the essential oils. Jumaa *et al*.^24^ and Weiss *et al*.^25^ reported a reduction in antimicrobial activity of nanoemulsions in comparison to uncapsulated active ingredients. Inappropriate formulation may lead to flocculation, Ostwald ripening or coalescence of the emulsion leading to a reduction in its stability and biological efficiency^26^. Consistent with our findings, Donsi *et al*.^27^ reported that high pressure homogenization as well as high shear homogenization results in decomposition of active constituents of essential oils particularly p-cymene, terpinenes, carveol, carvacrol and others. Interestingly, they reported a significant decrease in the amount of carvacrol as the process intensity increased from high shear homogenization to high pressure homogenization. This is in agreement with our GC-MS data which shows a large decrease in carvacrol upon nanoemulsion formation (Table 1).

As shown in Table 1 and Fig. 1C, the nanocapsules of the essential oil of *O. glandulosum* Desf. are characterized by the presence of 17 volatile constituents representing 98.50% of the total oil. Thymol (31.81%), carvacrol (28.45%), γ-Terpinene (15.26%) and p-cymene (9.09%) were the main constituents identified among the volatiles. These constituents are similar to those detected in hydrodistilled essential oil (Table 1). A significant quantitative difference was observed in the level of montoterpenes between hydrodistilled oil and nanocapsuled essential oil. Additionally, the majority of sesqueterpenes were not detected in the nanocapsules extract. This is obviously due to the processing technique which depends on homogenization at approximately 18000 rpm. However, it is less intense than the preparation of nanoemulsion. Hence, the volatiles profile of oil nanocapsules is still similar to that of the hydrodistilled oil and belongs to the thymol and / or carvacrol chemotype.

Further studies are necessary in order to explain the behavior of volatiles during different encapsulation processes and thereby evaluate the compatibility of the different encapsulation techniques for a set of natural oils and flavors.

### Effect of nanoencapsulation on the antioxidant activity of *O. glandulosum* Desf. oil

Antioxidant activities of hydrodistilled nanocapsules and nanoemulsion samples of *O. glandulosum* Desf. oil were evaluated using DPPH radical scavenging assay. Results are summarized in Table 2. The hydrodistilled oil of *O. glandulosum* Desf. had the lowest IC_50_ (4.22 mg/mL) which indicates a higher antioxidant activity, followed by nanocapsules of the oil (IC_50_ 57.51 mg/mL) and the nanoemulsions of the oil (IC_50_ 78.50 mg/mL). These values can be further explained by analyzing the total content of phenolics and flavonoids present in the samples and the differences in volatile compounds identified among the samples. Hydrodistilled oil, with the highest phenolic and flavonoid contents, was the most effective radical scavenger in comparison to synthetic antioxidant TBHQ, whereas nanoemulsions of the oil was the least effective (Table 2). Many effective phenolic components were detected in both hydrodistilled oil and its nanocapsules but very low amounts of phenolics, especially thymol and carvacrol, were present in oil nanoemulsions (Table 1). Naturally occurring phenolic compounds have been reported to be more effective as antioxidants with higher activities than synthetic ones^28^. However, the synergistic interaction between the phenolic content and the other components in the essential oil is also responsible for the high antioxidant activity^29^. The above results are consistent with the previous reports published by Sari *et al*.^1^ and Mechergui *et al*.^3^ who studied the antioxidant activity of *O. glandulosum* Desf. oil but not its encapsulated forms. Therefore, the results presented in this study suggests that the essential oil of *O. glandulosum* Desf. as well as its nanocapsules and nanoemulsions offer a cost-effective source of biological ingredients which can be used as antioxidants in food and pharmaceutical formulae instead of synthetic antioxidants.

**Table 2:** Antioxidant activity of HD, nanocapsules and nanoemulsion of *O. glandulosum* Desf. essential oil in comparison to the synthetic antioxidant TBHQ.

Material	IC_50_/mg/ml(DPPH)^a^	Total flavonoid content ^a^ mg CE/g	Total phenolic content ^a^ mg RE/g
		for 1 mg/ml	for 1 mg/ml
*O. glandulosum* Desf. HD oil	4.22 ± 0.13^a^	0.458 ± 0.02^a^	0.994 ± 0.01^a^
Nanocapsules of *O. Glandulosum* Desf. oil	57.51 ± 0.26^b^	0.312 ± 0.01^b^	0.756 ± 0.03^b^
Nanoemulsion of *O. glandulosum* Desf. oil	78.50 ± 1.13^c^	0.183 ± 0.01^c^	0.430 ± 0.09^c^
TBHQ	1.39 ± 0.12^d^	—	—

^a^Values represent averages ± standard deviations for triplicate experiments. Means with the same letter within the same row are not significantly different (*P* > 0.05).

### Morphological analyses

**Figure 2:**
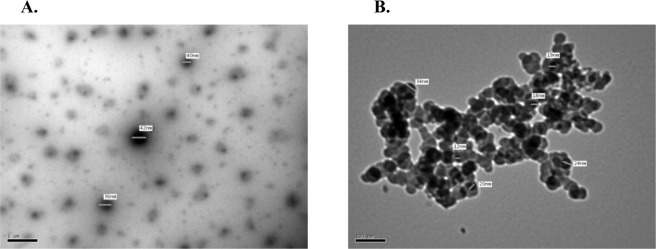
Transmission electron microscope (TEM) images: (**A**) nanoemulsion of *O. glandulosum* Desf. oil, and (**B**) nanocapsules of *O. glandulosum* Desf. oil.

Transmission electron microscopy (TEM) (Fig. 2) was used to investigate the morphology of the nanoparticles. Nanoemulsion particles appear dark and spherical in shape with an average diameter of 26.8 ± 0.11 nm (Fig. 2A). On the other hand, the size of nanocapsules was slightly bigger (34.65 ± 0.76 nm in diameter) due to the incorporation of oil droplets inside the sodium alginate wall (Fig. 2B). This is in agreement with the results published by Sotelo-Boyas *et al*.^30^ where they showed that the increase in nanocapsule size is related to encapsulation of the oil. The morphology of the outer surface of the capsule show the presence of the carrier agents with spherical shapes and smooth surfaces. The TEM image do not show any cracks or pores in the nanocapsules. As the bioactive constituents of *O. glandulosum* Desf. oil used in this work are highly volatile, the absence of cracks or pores on the surface of the nanocapsules represents a key feature, suggesting that it could protect volatiles against release, degradation or interaction with environmental factors.

### Effect of nanoencapsulation on the anticancer activity of *O. glandulosum* Desf. oil

**Figure 3:**
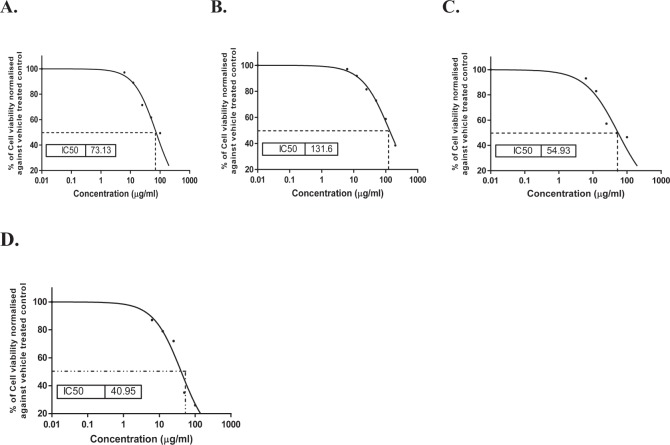
Evaluation of cell viability percentage of liver cancer cell line (Hep G2) post treatment (**A**) *O. glandulosum* Desf. HD oil, (**B**) nanoemulsion of *O. glandulosum* Desf. oil, and (**C**) nanocapsules of *O. glandulosum* Desf. oil compared with reference drug (**D**) 5-Fu using MTT assay.

**Figure 4:**
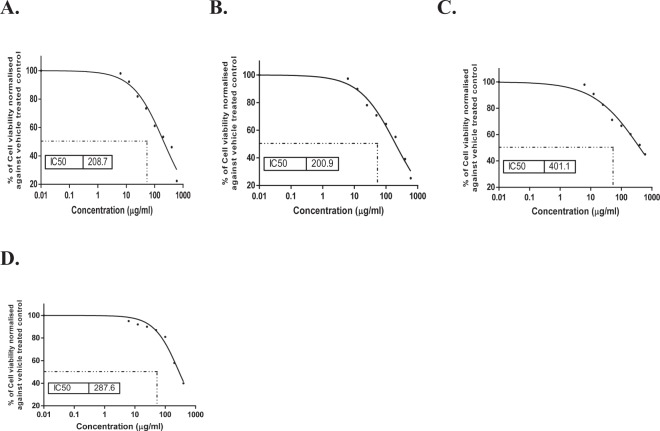
Evaluation of cell viability percentage of Healthy human hepatic cells (THLE2) post treatment (**A**) *O. glandulosum* Desf. HD oil, (**B**) nanoemulsion of *O. glandulosum* Desf. oil, and (**C**) nanocapsules of *O. glandulosum* Desf. oil compared with reference drug (**D**) 5-Fu using MTT assay.

The presumed cytotoxic effects of *O. glandulosum* Desf. Oil nanoemulsions and nanocapsules against Hep G-2 and THLE2 were investigated in this study, using MTT viability assay. 5-flurouracil was used as a reference drug. Figure 3 shows a reduction in cell viability percentage of liver cancer cell line (Hep G-2) after treatment with *O. glandulosum* Desf. Oil nanoemulsions and nanocapsules, as compared to treated THLE2 cells (Fig. 4). Nanocapsules of the oil exhibited the highest growth inhibitory activity against Hep G-2 cell line with the lowest IC_50_ (54.93 μg/mL) compared to 5-Fu (IC_50_ 40.95), followed by the hydrodistilled oil (73.13 μg/mL) whereas the nanoemulsions of the oil showed the least inhibition with IC_50_ (131.6 μg/mL). The lower IC_50_ values of Hep G-2 cells in comparison to THLE2 cells, showing the selectivity of the studied *O. glandulosum* Desf. oil as well as its nanoemulsions and nanocapsules.

Many bioactive compounds that are well–known as anticancer agents, including borneol, p-cymene, γ-terpinene, α-terpineol, caryophyllene, thymol, and carvacrol, were identified as major constituents in *O. glandulosum* Desf. oil as well as its nanoemulsions and nanocapsules^31^. However, quantitative differences exist in the volatile constituents detected (Table 1). This might be contributing to considerable differences in the anticancer and cytotoxic activities between *O. glandulosum* Desf. oil and its nanoparticles. According to Ozcan and Erdogan^29^, the essential oil of *Origanum onites* L. was found to be less toxic than its major components, thymol and carvacrol. Both phenols were the predominant components of nanocapsules of *O. glandulosum* Desf. oil (Table 1). Therefore, it can be concluded that the higher cytotoxicity of the nanocapsules can come from its phenolic components thymol and carvacrol regardless the total phenolic and flavonoid contents. The expected cytotoxicity mechanism of carvacrol, thymol and/or oregano essential oils, might be due to the induction of Glutathione S-Transferase (GST) which plays a major role in detoxifying carcinogens^23^. These findings suggest that, further studies will be necessary in order to reveal the correlation between antioxidant and anticancer activities along with the synergetic and/or antagonistic among constituents of botanical extracts.

## Conclusions

The effect of nanometric delivery systems of *O. glandulosum* Desf. oil on antioxidant and anticancer activities as well as volatile constituents was investigated as a method to employ compounds of botanical origin for application in both food industry and cancer treatment. Using of high - speed and high - pressure homogenization techniques during encapsulation affected dramatically the volatile constituents of delivery systems in comparison to the original oil and therefore the biological activities. Further studies are necessary in order to explain the behavior of chemical constituents during different encapsulation processes and thereby evaluate the compatibility of the different encapsulation techniques for a set of natural oils and flavors.

## Data Availability

All data generated or analyzed during this study are included in this article.
